# Assessing the health risks of chemicals in a company supplying chemicals to drilling rigs in Southern Iran using COSHH, SHEM-SAM, and SQRA methods

**DOI:** 10.3389/fpubh.2024.1395695

**Published:** 2024-09-26

**Authors:** Sajad Astani, Bahareh Lorestani, Mehrdad Cheraghi, Maryam Kiani Sadr

**Affiliations:** Department of the Environment, College of Basic Sciences, Hamedan Branch, Islamic Azad University, Hamedan, Iran

**Keywords:** chemical exposure, toxic material, chemical industry, risk assessment methods, occupational health and safety, drilling industries

## Abstract

Chemical industries are among the process industries and cause many risks. The present research aimed to analyze the health risks of a chemical warehouse of drilling rigs chemical Supply Company based on COSHH (Control of Substances Hazardous to Health), CHEM-SAM (Chemical Risk Management Self-Assessment Model), and SQRA (Subjective Quantified Risk Assessment) methods in 2021. The information was collected based on document review, MSDS of chemicals, processes, employees, and chemical exposure. Flammability, toxicity, allergy-causing, corrosivity, reactivity, LD50, and permissible thresholds of chemicals were also collected. The present research results showed that out of 59 main chemicals in the operational processes of the chemical warehouse of drilling rigs, 14 chemicals are flammable, 22 cause allergy responses, and three can cause death if inhaled. According to the results of the CHEM-SAM method, the employees and people outside the organization are at medium and low chemical risk based on the current management of the chemical warehouse, respectively. The results of the COSHH health assessment showed that chemicals had low, medium, high, and very high risk in 31, 13, 12, and 3 cases, respectively. The high-risk cases consisted of Ammonium Nitrate، Caustic Soda, and Poly.Aluminum.Chloride. Health risk assessment was also performed using the SQRA method, and results showed that chemicals have a very low, low, medium, high, and very high risk in 27, 12, 8, 9, and 3 cases, respectively. The results showed that the adverse health effects of chemical exposure in the drilling industry are alarming. Employees of different sectors of oil and gas industries are exposed to chemicals.

## Introduction

Every year, organizations face billions of dollars of losses in human, equipment, and reputational losses due to work-related accidents and injuries (1). A significant part of these accidents and injuries are related to the chemical industry (2). Chemical exposure to various processes of chemical industries can have various adverse health effects on humans (3–5). Research has shown that more than 25 million workers are exposed to occupational hazards such as dust, various toxins, and noise from the work environment (6). Drilling oil and gas wells are one of the stages of the oil process (7, 8). Many chemicals are used for different purposes in the well drilling process, such as cooling and lubrication of the drill, pressure control, and integration of the well wall (9–11). Such chemicals include barite, caustic, biocide, soda, pipe lax, starch, foam, and even diesel (12, 13). Depending on the characteristics of the well, different geological, climatic, and geographical components, different chemicals are used for the circulating fluid in the drilling well (14, 15). The effects of these substances have been investigated in various researches. Caganova et al. (16) concluded in a study that caustic exposure has significant effects on changes in the acidity characteristics of workers’ bodies. In a study, Kim and Kim (17) investigated the effects of biocide exposure on workers’ skin. The results of this study showed that long-term exposure to this substance, in addition to causing burns, has significantly increased the odds of skin cancer. Ibrahim et al. (18) also stated that calcium chloride had adverse effects on the sexual characteristics of workers. These studies show the necessity of a health-risk study in the storage centers of these chemicals. Health-risk assessment of chemicals helps workers to identify and eliminate the hazards of their work environment to have a safe work environment (19, 20). There are several methods to identify and assess health risks (21, 22). In modern health risk assessment methods, several components are involved in determining the risk level (23). One of the new methods of assessing the health risks of chemicals is COSHH. COSHH regulations require all employers to assess the risks to health arising from hazardous substances in the workplace. Factors such as the possibility of removing and replacing hazardous substances, the existence of a material safety information sheet, process description, risk classification, type of effect, work environment limitations, possible control facilities, and the priority of dealing with hazardous substances are presented in this method. This method was presented by the Institute of Chemical Studies of the European Parliament in 2006 (24). Another relatively new method in health risk assessment is SQRA, which is a semi-quantitative method for health risk assessment of hazardous chemicals. In this method, the LD_50_ index is used for skin absorption, respiratory, vital organs, and surface absorption. Two factors are important in this method, the hazard rate and exposure rate (25, 43). Another health risk assessment method used in the present research is the CHEM-SAM technique. This technique was designed by the International Chemical Threat Reduction Program Department of Sandia Laboratories in the United States (26). This method answers three basic questions: 1. what can happen? 2. How much is the likelihood of an accident? 3. If it happens, what are the consequences? This method is based on several steps: determining the properties of chemicals, defining the potentially harmful properties of chemicals, calculating the safety risk of chemicals, and determining risk acceptability (27). These chemical risk assessment methods have been used in various research. Tian et al. (18) called the COSHH method a desirable method for risk assessment in the chemical industry. Lee et al. (19) also used the COSHH method to assess the health risks of methyl chloride, isopropanol, and acetone. Karimi Zeverdegani et al. (30) also confirmed the reliability of the CHEM-SAM method based on semi-quantitative models. Overall, one of the hazards of the oil and gas industry is chemicals (31). There is a possibility of various injuries in these industries due to the direct exposure of employees to various chemicals. Considering the high potential of damage caused by exposure to these substances and their wide use in Iranian drilling industries, it seems necessary to conduct comprehensive research on the risk assessment of exposure to these substances using modern assessment techniques.

Chemical supply companies for oil and gas well drilling rigs are among the chemical distribution centers that have received less attention and study. These companies are mainly active in oil-producing countries. Conducting a complete study of the risk potential of chemical substances for employees and other people who are exposed to its activity is considered research innovation. Therefore, the health risk caused by exposure to these substances has been investigated in the present study using COSHH, SQRA, and CHEM-SAM methods.

## Materials and methods

MI Services, a chemical supply company for drilling rigs, is located in the oil-rich areas of southwestern Iran. This company has three chemical warehouses used in the drilling industry with a total area of 80,000 m^2^. On average, 40 tons of chemicals enter and leave warehouses every day. The loading and unloading process of 500 kg and 50 kg packs is carried out by forklifts and drums by warehouse workers manually, respectively. A total of 38 warehouse workers are constantly in direct contact with these substances.

The current descriptive-analytical research was conducted to identify and assess the health risks of raw substances in this company in 2021. After preparing a list of all the raw substances, physicochemical properties such as toxicity, flammability, and reactivity were prepared through the preparation of MSDS or, if necessary, international references and standards such as NIOSH and OSHA. Considering that the health risk levels are based on the employees’ exposure level, related information was also collected by field method. This information included the employees of different departments of the warehouses, working hours, operation processes, and the level of exposure to chemicals. Finally, the health risk of raw substances was identified and assessed using COSHH, CHEM-SAM, and SQRA methods.

### Health risk assessment using COSHH method

The instructions of the Organization for Control of Substances Hazardous to Health for chemical compounds have been classified in the two parameters of consequence and exposure likelihood in the standard matrix and questionnaire, as very low, low, medium, high, and very high risk (44). The exposure likelihood was calculated by expert observations of the work environment, relevant measurements, and by considering parameters such as the type and amount of chemicals used in chemical warehouses. The assessment matrix of the consequence severity and the exposure likelihood in the COSHH method is presented in Table 1 and the risk classification based on the risk priority number is presented in Table 2.

**Table 1:** Matrix of severity and risk of exposure to chemicals in COSHH method (41).

Risk of exposure				
Consequence of hazard	Unlikely (1)	Possible (2)	Likely (3)	Very Likely (4)
Minor (1)	1	2	3	4
Moderate (2)	2	4	6	8
Serious (3)	3	6	9	12
Very Serious (4)	4	8	12	16
Extreme (5)	5	10	15	20

**Table 2:** Classification of health risks in COSHH method based on priority number (41).

	Risk rating
Low	1–5
Medium	6
High	8–10
Very High	12–20

### Health risk assessment using the SQRA method

There are two important factors in this method: Hazard rate (HR) and exposure rate (ER). After identifying the chemical raw substances in the company’s warehouses, HR was determined using information on acute toxicity values, LD_50_ and LC_50,_ or through the toxic effects of chemicals defined in tables. The second factor is the exposure rate (ER), which is obtained using the following two ways:

First: Based on actual exposure levels when air monitoring results are available. The weighted average weekly exposure is calculated using the Equation 1:

(1) $$E=\frac{F\times D\times M}{W}$$

Where; E: Weekly exposure (ppm or mg/m^3^), F: Frequency of exposures per week, M: Exposure level (ppm or mg/m^3^), W: Average weekly working hours (40 h), D: Average exposure time in hours.

The calculated E is compared with the occupational exposure limit (OEL) and then the exposure rate is determined according to Table 3.
When exposure to two or more chemicals with similar effects occurs, combined exposure is taken into consideration.
Second: Using ER index: when air monitoring results are not available, the exposure degree can be obtained using the exposure index (EI) and by the Equation 2:

(2) $$R={\left[\frac{E}{1}\times \frac{E}{2}\times \ldots .\times \frac{E}{n}\;\right]}^{\frac{1}{2}}$$

Where *n* is the number of exposure indices used and EI is the exposure index.

**Table 3:** Chemical exposure rate in SQRA method (42).

Exposure level	E/OEL
1	Less than 0.1
2	0.1–0.5
3	0.51–1
4	1.01–2
5	More than 2

The EI (exposure index) is determined according to the exposure factors defined in the tables. Finally, the hazard rate is calculated by calculating ER and HR through the Equation 3:


(3) $$\mathrm{SQRA = (HR \times ER)}\;\frac{1}{2}$$

Risk is obtained in five levels: insignificant (0–0.5), low (0.51–1), medium (1–2), high (2–3), and very high (>3).

### Health risk assessment using CHEM-SAM method

**Figure 1:**
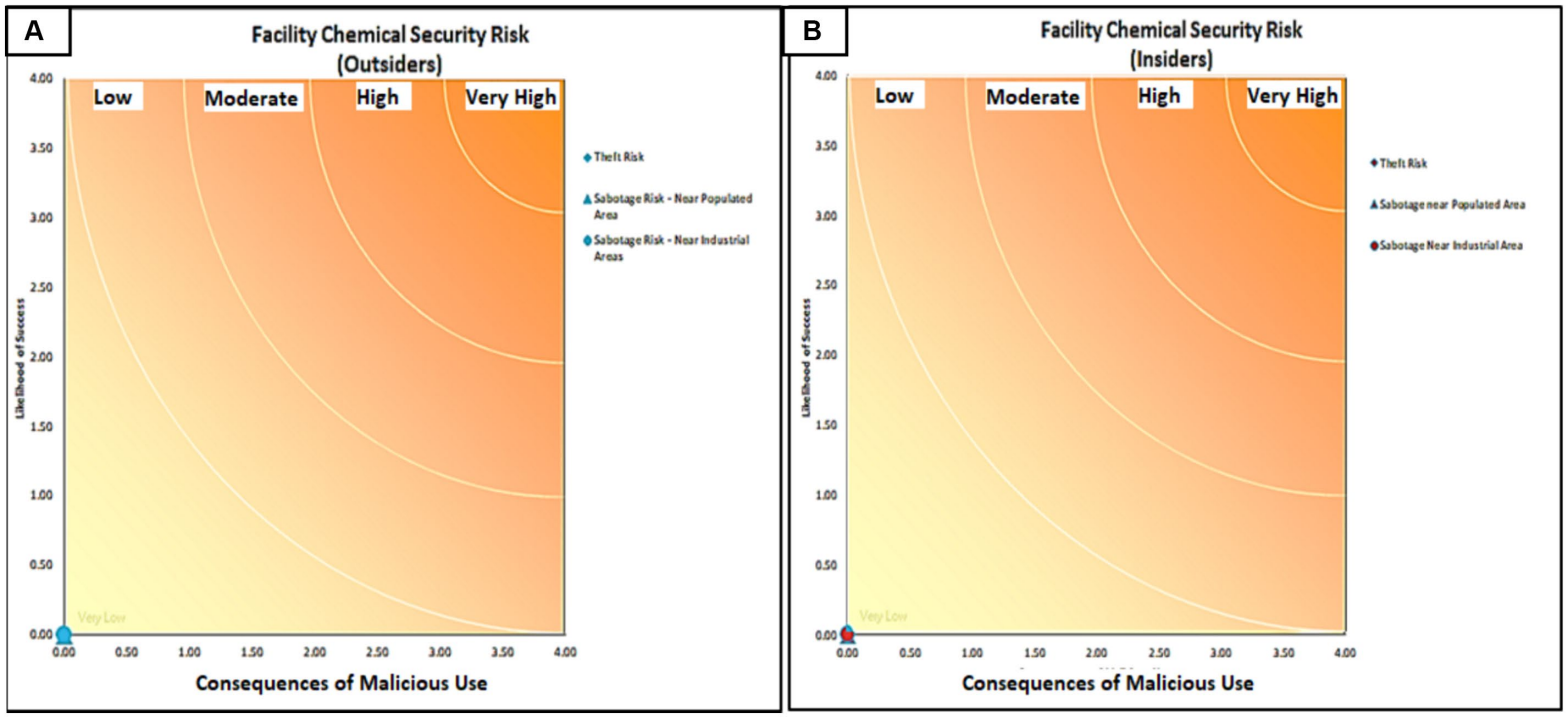
Output for assessing the lethality level of chemical risk (A: Outside the enclosure; B: Inside the enclosure)-2021.

This method, which was designed by the International Chemical Threat Reduction Department of Sandia National Laboratories in United States, is based on several steps. Defining the potentially harmful properties of chemicals, calculating the safety risk of chemicals, and determining risk acceptability. This method contains 63 questions on variables such as the storage conditions of and substance transfer, toxicity, flammability, volatility, physical form, route of entry into the body, type of exposure, safe packaging conditions of the substance, control conditions in the desired environment, labeling, control conditions outside the work environment, warning items, training to control conditions outside the work environment, warning substances, substance destruction conditions, management, and planning that are designed in Excel software. Each variable includes related questions that are answered based on four options. After answering all the questions in the Excel software, the risk level is determined. This method consists of five levels of risk: very low, low, medium, high, and very high (26) (Figure 1).

## Results

To analyze the health risks of MI Services company’s raw substances, a list of chemicals used in warehouses was prepared. This list contains 59 chemicals (Table 4).

**Table 4:** List of chemicals identified in the drilling rig chemical supply company-2021.

Salt	49	VG 69 (DRM)	33	DCP 208 (GLYDRILL MC)	17	Acetone	1
Sodium bicarbonate	50	M-I PAC-R	34	GLYCOLE HC	18	Ammonium nitrate	2
Sodium silicate	51	M-I PAC-UL	35	Drilling detergent	19	Antifoam silicon	3
Soda ash	52	MICA-M	36	Fiber lock	20	Barite	4
Spersene	53	Natural Gum	37	H2S Scavenger	21	Bentonite	5
MI Starch	54	NUT PLUG-C	38	Hematite	22	Bit Lube	6
STARCH Potato	55	OS1-L	39	KCL B.B	23	Biocide	7
STARCH Wheat	56	OYSTER SHELL-XC	40	KCL sxs	24	Calcium bromide	8
STARCH HT	57	P.T.S. 200	41	KWICK SEAL-M	25	Calcium carbonate	9
Xanthan gum	58	PIPE LAX	42	Lime	26	Calcium chloride	10
Toluene	59	POLY.ALUMINUM.Chloride	43	Mg oxide	27	Caustic soda	11
		Soybean oil, crude	44	M-I coat	28	Cement	12
		Xylenes	45	M-I DME	29	Citric acid	13
		Naphthalene	46	M-I MUL	30	CMC-LV	14
		Benzene	47	O.B.M FLC	31	CONQOR-404(WS)	15
		Safe Cide	48	VG 69(MIOVIS)	32	MI COR-DPE 55-MICOR(OS)	16

### Results of the health risk assessment using of COSHH method

The risk priority in this method is the product of two risk severity components (the severity of the chemical risk) and the risk likelihood (the level of chemical exposure). The severity component is classified into five levels: low, medium, serious, very serious, and severe. The likelihood component is also classified into four levels: unlikely, possible, likely, and highly likely. Part of the results of the COSHH-based health risk assessment of chemicals are presented in Table 5.

**Table 5:** Results of chemical risk assessment of chemicals in COSHH method in the drilling rig chemical supply company-2021.

Row	Chemical name	Likelihood of exposure by employees (minutes per day)	Number of employees exposed to the chemical	Chemical hazard severity score	Chemical exposure likelihood score	Risk priority number	Risk acceptance
1	Acetone	10	10	4	3	12	H
2	Ammonium nitrate	10	10	5	4	20	VH
3	Antifoam silicon	60	10	1	3	3	L
4	Barite	480	10	2	4	8	M
5	Bentonite	480	10	4	4	16	H
6	Bit lube	60	10	3	3	9	M
7	Biocide	180	10	4	4	16	H
8	Calcium bromide	60	10	3	3	9	M
9	Calcium carbonate	60	10	3	3	9	M
10	Calcium chloride	60	10	4	4	16	H
11	Caustic soda	180	10	4	5	20	VH
12	Cement	360	10	2	5	10	M
13	Citric acid	60	10	3	4	9	M
14	CMC-LV	100	10	2	3	6	L
15	CONQOR-404(WS)	50	10	1	3	3	L
16	MI COR-DPE 55-MICOR(OS)	50	10	2	3	6	L
17	DCP 208 (GLYDRILL MC)	80	10	2	4	8	M
18	Glycole HC	100	10	2	4	8	M
19	Drilling detergent	180	10	3	4	12	H
20	Fiber lock	180	10	1	4	4	L
21	H2S scavenger	0	10	5	1	5	H
22	Hematite	100	10	4	4	16	H
23	KCL B.B	120	10	1	4	4	L
24	KCL sxs	120	10	1	4	4	L
25	KWICK SEAL-M	60	10	1	3	3	L
26	Lime	420	10	1	5	5	L
27	Mg Oxide	60	10	1	3	3	L
28	M-I Coat	60	10	1	3	3	L
29	M-I DME	60	10	1	3	3	L
30	M-I MUL	60	10	3	3	9	M
31	O.B.M FLC	10	10	2	2	4	L
32	VG 69 (MIOVIS 69)	60	10	1	3	3	L
33	VG 69 (DRM)	60	10	1	3	3	L
34	M-I PAC-R	120	10	1	4	4	L
35	M-I PAC-UL	120	10	2	4	8	M
36	MICA-M	120	10	1	4	4	L
37	NATURAL GUM	120	10	1	4	4	L
38	NUT PLUG-C	120	10	1	4	4	L
39	OS1-L	120	10	1	4	4	L
40	OYSTER SHELL-XC	120	10	2	3	6	L
41	P.T.S. 200	120	10	2	3	6	L
42	PIPE LAX	30	10	5	2	10	H
43	POLY.ALUMINUM.Chloride	20	10	4	5	20	VH
44	Poly Amine	10	10	4	3	12	H
45	Xylenes	430	4	4	3	12	H
46	Naphthalene	150	10	4	3	12	H
47	Benzene	150	10	3	4	12	H
48	Safe Cide	180	10	1	4	4	L
49	Salt	420	10	1	5	5	L
50	Sodium bicarbonate	60	10	3	3	9	M
51	Sodium silicate	60	10	2	3	6	L
52	Soda ash	180	10	2	4	8	M
53	Spersene	40	10	1	3	3	L
54	MI STARCH	420	10	1	5	5	L
55	STARCH Potato	420	10	1	5	5	L
56	STARCH WHEAT	420	10	1	5	5	L
57	STARCH HT	420	10	1	5	5	L
58	Toloene	10	10	4	3	12	H
59	Xanthan gum	50	10	2	2	4	L

The likelihood of exposure is calculated based on the total time of employees’ exposure to chemicals, in minutes per day. The exposure time has been continuous in some cases and interrupted in some cases. The Chemical hazard severity score component was considered based on the degree of danger of each chemical in the MSDS, which is based on international authorities such as NIOSH, ACGIH, and OSHA. Considering that the basis for calculating the risk priority is the level of exposure, some hazardous chemicals that employees have a low level of exposure to are not estimated as a priority.

The results of the COSHH assessment showed that 31 chemicals (52.5%) have low risk, 13 chemicals (22.03%) have medium risk, 12 chemicals (20.3%) have high risk and 3 chemicals consist of Ammonium Nitrate، Caustic Soda, and Poly. Aluminum.Chloride (5.08 %) has a very high risk. The highest risk levels were related to ammonium nitrate and benzene with a priority number of 20. The risk priority number for materials including cement, Poly Amine, Pipe Lax, and Hematite has also been at a high level.

### Results of the health risk assessment using CHEM-SAM method

**Figure 2:**
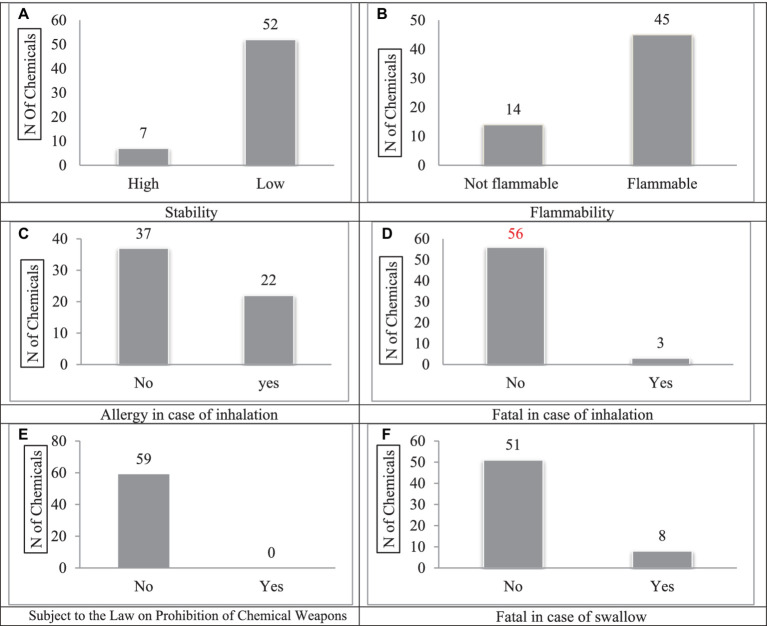
Classification of chemicals based on some of the components studied in the CHEM-SAM method-2021. **(A)** Stability, **(B)** Flammability, **(C)** Allergy in case of inhalation, **(D)** Fatal in case of inhalation, **(E)** Subject to the Law on Prohibition of Chemical Weapons, **(F)** Fatal in case of swallow.

The CHEM-SAM-based health risk assessment relies on the nature of the data and the management of chemicals. In this assessment method, 10 components were evaluated separately for each chemical and 53 components were evaluated in general for all chemicals. The assessment output in this method consists of five levels of very low, low, medium, high, and very high risk for employees and people outside the organization. The results of the health risk assessment of the studied chemicals are shown in the graphs in Figure 2.

According to the results, 7 chemicals, including bentonite, calcium chloride, cement, drilling detergents, biocide, hematite, and KCL BB, have high stability in the environment (Figure 2A).

**Figure 3:**
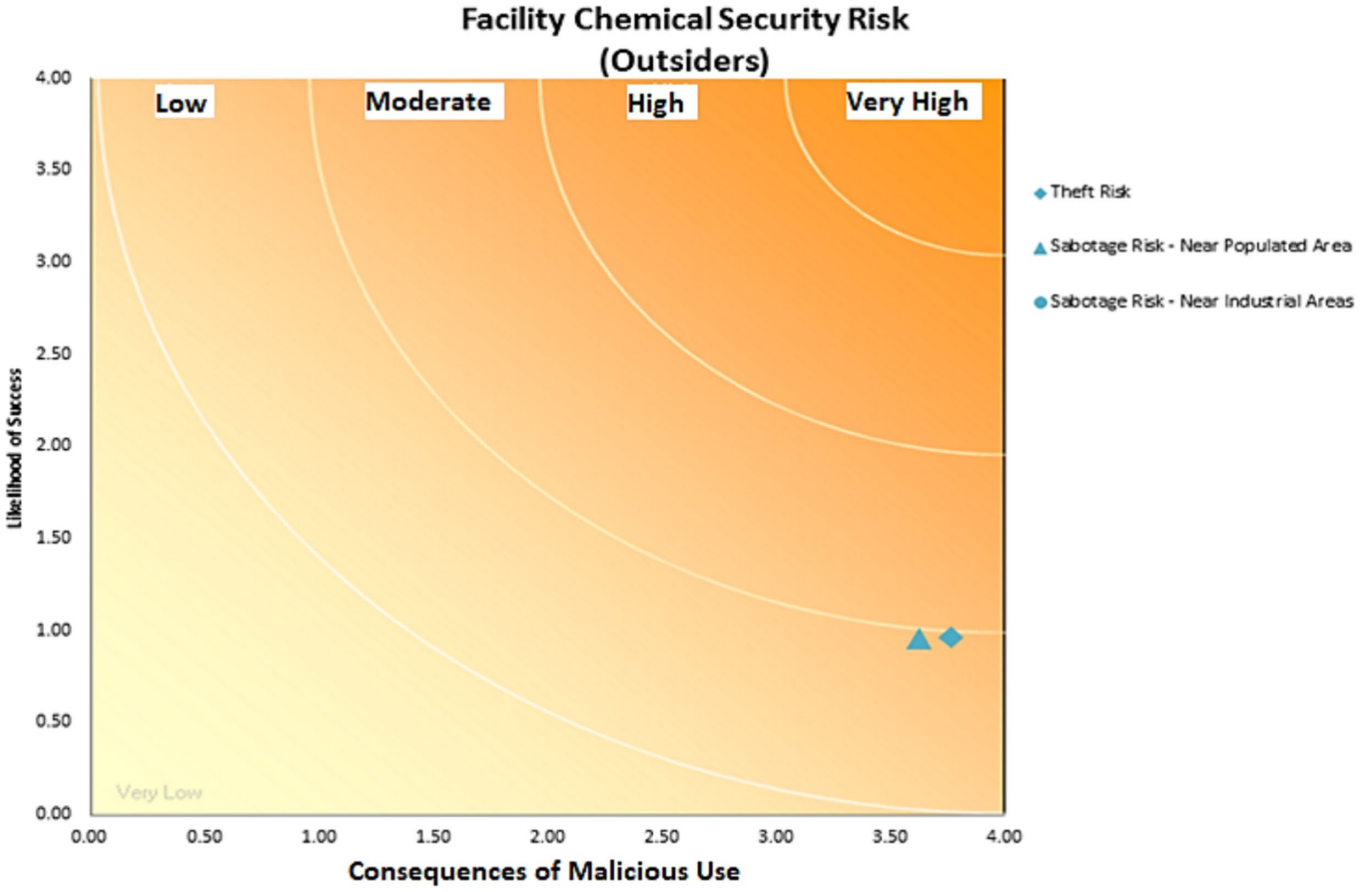
Chemical safety risk of equipment for people outside the drilling rig chemical supply company-2021.

**Figure 4:**
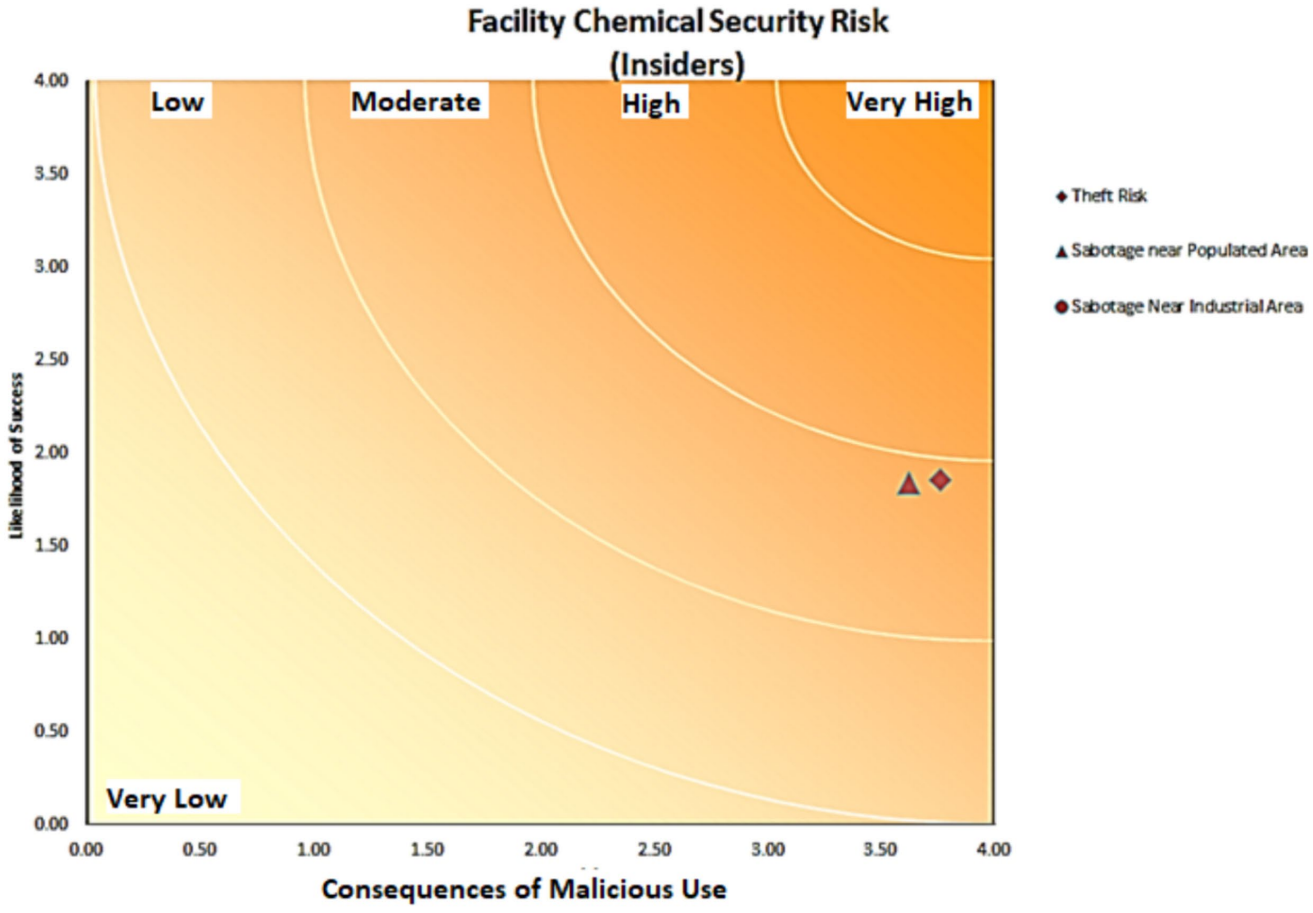
Chemical safety risk of equipment for people inside the drilling rig chemical supply company-2021.

A total of 14 chemicals in MI company’s chemical warehouse are flammable (23.7%) and 45 chemicals are non-flammable (76.3%) (Figure 2B). Of the 59 studied chemicals, 22 cause allergic responses (in the case of inhalation 37.3 % of the total chemicals) (Figure 2C). Also, a total of 23 cases (39%) of the chemicals used in MI Company cause some kind of chronic effect and 18 cases (30.5%) of them induce an acute effect. Moreover, seven chemicals (11.8%) are stable in the environment. The three substances including biocide, hydrogen sulfide, and pipe lax (5%) can lead to death if inhaled (Figure 2D). The results of Chem-Sam-based chemical risk assessment are shown in Figures 3, 4. The results also showed that no chemicals stored in the company’s warehouse are on the list of the Organization for the Proliferation of Chemical Weapons (Figure 2E). A total of 8 chemicals (13.5%) used in the company’s chemical warehouse, including acetone, ammonium nitrate, bentonite, biocide, calcium chloride, caustic, pipe lax, and toluene, lead to death if swallowed (Figure 2F). The results show the exposure of employees to dangerous chemicals, with various consequences in this Company Supplying Chemicals.

**Figure 5:**
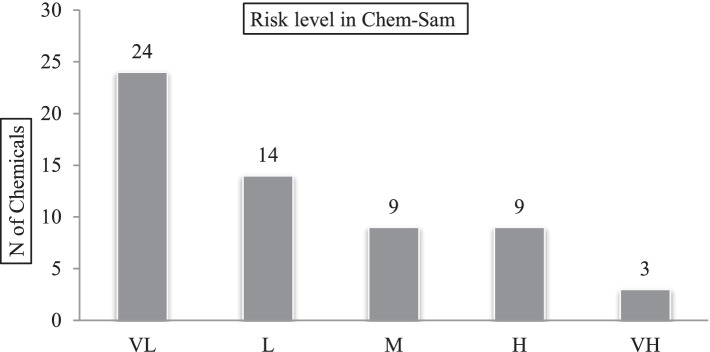
Frequency distribution of chemical risk levels based on Chem Sam method in the drilling rig chemical supply company-2021.

According to the CHEM-SAM method, it was found that employees are at a medium risk of chemicals based on the current management of chemicals in the chemical warehouse of the MI company (Figure 3) and people outside the organization are at a low risk (Figure 4). It was also found that a total of chemicals caused a very low, low, medium, high, and very high risk in 24 (40.67%), 14 (23.7%), 9 (15.2%), 9 (15.2%) and 3 cases (5%), respectively (Figure 5).

### Results of health risk assessment using SQRA method

The summary of the results of the health risk assessment of chemicals used in the MI Company is presented in Table 6. SQRA consists of two important factors: Hazard rate (HR) and exposure rate (ER). OEL/TWA and TLV indices have been used for the exposure level. The average minutes of daily exposure were also converted to hours per week. ER was calculated using Equation 2 and, finally, the risk priority of chemicals was calculated by multiplying it by the HR.

**Table 6:** Results of chemical risk assessment of chemicals in SQRA method in the drilling rig chemical supply company—2021.

Risk level	HR	ER/OEL	ER	OEL/TWQ/TLV	Average weekly exposure in hours (D)	Chemical	Row
H	4	0.00252	0.63	250	0.833	Acetone	1
VH	5	0.5	5	10	0.833	Ammonium nitrate	2
VL	1	0	5	-	5	Antifoam silicon	3
L	2	0	0.63	-	40	Barite	4
H	4	0.00188	1.88	1,000	40	Bentonite	5
M	3	0	0.63	-	5	Bit lube	6
H	4	0.000525	0.63	1,200	15	Biocide	7
M	3	0	0.63	-	5	Calcium bromide	8
M	3	0	1.88	-	5	Calcium carbonate	9
H	4	0.025	3.75	150	5	Calcium chloride	10
H	4	0.0126	0.63	50	15	Caustic soda	11
L	2	0	1.04	-	30	Cement	12
M	3	0.0065	0.52	77–92	5	Citric acid	13
L	2	0	0.52	-	8.333	CMC–LV	14
VL	1	0	0.83	-	4.167	CONQOR—404(WS)	15
L	2	0	1.04	-	4.167	MI COR-DPE 55-MICOR(OS)	16
L	2	0	1.88	-	6.667	DCP 208 (GLYDRILL MC)	17
L	2	0	1.88	-	8.333	GLYCOLE HC	18
M	3	0	0	-	15	Drilling detergent	19
VL	1	0	1.04	-	15	Fiber lock	20
VH	5	0.089286	1.25	14	0	H2S Scavenger	21
H	4	0.000625	1.25	2,000	8.333	Hematite	22
VL	1	0	0.63	…	10	KCL B.B	23
VL	1	0	4.38	-	10	KCL sxs	24
VL	1	0	0.63	-	5	KWICK SEAL-M	25
VL	1	0	0.63	-	35	Lime	26
VL	1	0	0.63	-	5	Mg Oxide	27
VL	1	0	0.63	-	5	M-I COAT	28
VL	1	0	0.1	-	5	M-I DME	29
M	3	0	0.63	-	5	M-I MUL	30
L	2	0	0.63	-	0.833	O.B.M FLC	31
VL	1	0	1.25	-	5	VG 69 (MIOVIS 69)	32
VL	1	0	1.25	-	5	VG 69 (DRM)	33
VL	1	0	1.25	-	10	M-I PAC-R	34
L	2	0	1.25	-	10	M-I PAC-UL	35
VL	1	0	1.25	-	10	MICA-M	36
VL	1	0	1.25	-	10	NATURAL GUM	37
VL	1	0	1.25	-	10	NUT PLUG-C	38
VL	1	0	1.25	-	10	OS1-L	39
L	2	0	0.31	-	10	OYSTER SHELL-XC	40
L	2	0	0.21	-	10	P.T.S. 200	41
VH	5	0.01	0.1	10	2.5	PIPE LAX	42
H	4	0.0094	1.88	100–300	1.667	POLY.ALUMINUM.Chloride	43
H	4	0.0235	1.88	40–100	0.833	POLY AMINE	44
H	4	0.28	42	-	5	Xylenes	45
H	4	0	1.88	10	15	Naphthalene	46
H	3	0	4.38	2.5	15	Benzene	47
VL	1	0	0.63	-	15	Safe Cide	48
VL	1	0	0.63	-	35	Salt	49
M	3	0	1.88	-	5	Sodium bicarbonate	50
L	2	0	0.42	-	5	Sodium silicate	51
L	2	0	4.38	-	15	Soda ash	52
VL	1	0	4.38	-	3.333	Spersene	53
VL	1	0	4.38	-	35	MI Starch	54
VL	1	0	4.38	-	35	STARCH Potato	55
VL	1	0	0.1	-	35	STARCH Wheat	56
VL	1	0	0.52	-	35	STARCH HT	57
H	4	0.018794	0	199	0.833	Toluene	58
L	2	0	5	-	4.167	Xanthan gum	59

HR values are based on the severity of the hazard of each chemical, regardless of the level of employee exposure. By calculating the ratio of ER to OEL/TLV, the risk of exposure to hazardous substances can be quantitatively evaluated. This ratio shows that a percentage of the permissible threshold for exposure to a hazardous substance in the work environment is affected. By analyzing this ratio and checking the allowed thresholds, it is possible to prioritize.

**Figure 6:**
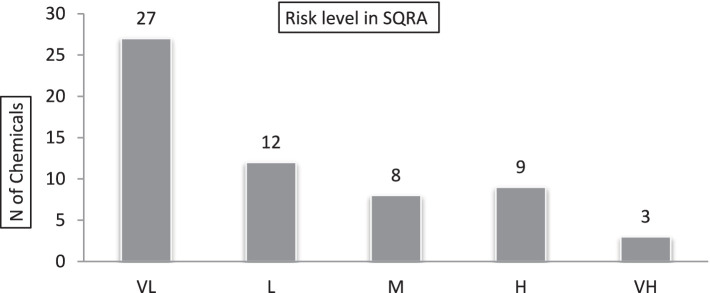
Frequency distribution of chemical risk levels based on SQRA method in the drilling rig chemical supply company-2021.

The frequency distribution of the risk of chemicals is presented in Figure 6. These results show that chemicals caused a very low, low, medium, high, and very high risk in 27 (46%), 12 (20%), 8 (14%), 9 (15%) and 3 cases (5%), respectively (Figure 6). The results of the risk assessment using the SQRA method also showed the risk of exposure to Ammonium Nitrate, Caustic Soda, and Poly.Aluminum.Chloride compounds are at a very high level.

Chemical risk assessment methods are based on indicators, so different results can be expected. Adaptation of the results is a method to measure the desirability of any evaluation method.

Kendall’s rank correlation coefficient was used to investigate the correlation between the results of chemical health risk assessment methods (COSHH, CHEM-SAM, and SQRA) used in MI Company’s chemical warehouse. These results showed a significant correlation between the results of SQRA and CHEM-SAM methods (Sig = 0.756). Kendall’s rank correlation coefficient was calculated to be 0.756 (Table 7). However, there was no significant correlation between the results of the COSHH with the CHEM-SAM and SQRA methods (*p* ≥ 0.05), which is probably due to the different priority classification of chemicals in the COSHH method with the CHEM-SAM and SQRA methods.

**Table 7:** Kendall coordination and correlation coefficient for comparison of COSHH, CHEM-SAM, and SQRA methods—2021.

			COSHH	SQRA
Kendall’s correlation	Chem_Sam	Correlation coefficient	0.588*	0.756**
		Sig. (two-tailed)	0.040	0.000
		*N*	75	59
	COSHH	Correlation coefficient	***	0.478
		Sig. (two-tailed)		0.063
		*N*		59

## Discussion

Oil industries, which are among the process industries, cause many risks and can lead to disastrous and irreparable consequences. These industries consist of four main characteristics including the quantity and quality of energy, low flexibility, high complexity, and high energy (32). Workers working in industrial workplaces, such as the chemical storage area of drilling rigs, are the most important group exposed to hazardous chemicals and their health should be protected. The methods used in the current research are based on the analysis and assessment of chemicals used in the chemical warehouse of the drilling industry. The results of the research showed that some of the 59 main chemicals in the chemical warehouse of the drilling rig require special management to reduce the level of health risk. Considering these results, there is a relatively high potential for injuries caused by exposure to chemicals in MI and the prevalence of some chronic symptoms such as respiratory problems and skin allergies.

Among the substances that can lead to allergic skin reactions, cement, calcium chloride, Drilling Detergent, and Caustic Soda can be mentioned. Biocide, which is used to eliminate microorganisms in drilling rig environments, contains chlorine compounds, formaldehyde, ammonium compounds, and sodium hypochlorite (33). The level of exposure risk to Biocide has been assessed as high in the SQRA method, with an average weekly exposure of 5 hours for warehouse personnel. The most significant consequence of exposure to Biocide is respiratory infections. Ammonium nitrate is another chemical compound present in the chemical storage for drilling rigs. It is used for flow control in oil wells. Its effects include respiratory irritation, gastrointestinal damage, and long-term damage to the nervous system (34). The present research indicates that the level of exposure risk to this chemical is very high based on the risk assessment.

Exposure to benzene, toluene, xylene, and naphthalene in the gas phase is another result of the present study. Benzene has been identified as a known carcinogen. Benzene can induce cytogenetic changes such as aneuploidy, gene expression alteration (128), DNA methylation (129), and displacement, leading to the production of carcinogenic proteins (35). Vital genes may also be targeted through gene mutation and/or epigenetic alterations. Additionally, toluene and xylene compounds have adverse effects on the nervous, respiratory, and immune systems. At high doses of toluene, human motor activity has been observed significantly lower than pre-exposure levels (36). According to the results of the present study, the level of exposure to these compounds for warehouse and laboratory staff was found to be high.

The harmful factors in the workplace were measured twice a year. Therefore, considering the nature of the organization’s activity and high exposure to chemicals, it is recommended that in addition to conducting this process every month, equipment for continuous testing of hazardous substances and their concentration in the environment should be prepared by the company. Also, part of the toxic and hazardous emissions from chemicals such as acids, polyamine, ammonium nitrate, and pipe lax are caused by leakage from packaging and drums. The poor packaging quality of chemicals sometimes leads to leakage and some injuries. Increasing the packaging inspection and packaging quality control of substances entering the chemical warehouses of drilling rigs is a suggestion that can prevent some damage. Liu et al. (37) have recommended the labeling of chemicals with flammability and reactivity characteristics and separate storage. Other causes of accidents include human errors during the transportation of chemicals by hand or forklift that requires the development of a response plan in emergencies. Traffic in chemical warehouses (open and isolated warehouses) must be in full compliance with safety standards. The use of gloves, special coveralls, protective glasses, and masks for the warehouse employees when working with chemicals and working with chemicals that have respiratory effects in an isolated room with a negative pressure of 2.5 pascals is one of the things suggested by Bergkamp and Abelkop (38).

Mechanical ventilation in the production area following the ACGIH standard is also one of the other recommendations related to the storage of chemicals.

Determining work shifts is one of the effective solutions to prevent chemical accidents. Bhusnure et al. (39) mentioned short-term work shifts as one of the effective solutions for preventing injuries caused by exposure to chemicals. OEL/TWA and TLV standards are used to determine work shifts. Sharma et al. (40) referred to waste contaminated with chemicals (such as napkins, cloth, cotton, etc.) as one of the factors influencing the chemical contamination of warehouses and laboratories containing chemicals, which requires management. Adjusting the ambient temperature while working with some substances such as chloroform reduces its emission in the air.

## Conclusion

The results of the present research showed that the adverse health effects of chemical exposure in the drilling industry are at a worrying level. The results of chemical exposure risk assessment using SQRA, CHEM-SAM and COSHH methods showed that there is a high potential for health consequences from exposure to compounds such as ammonium nitrate, caustic soda and poly-aluminum chloride, benzene, xylene and toluene for There are staff. It is suggested that the techniques used in the present research should be used in other sectors of these industries and its results should be used in health risk management and control programs.

## Data Availability

The original contributions presented in the study are included in the article/supplementary material; further inquiries can be directed to the corresponding author/s.
